# Factors associated with citation rate of randomised controlled trials in physiotherapy

**DOI:** 10.1186/s40945-015-0009-6

**Published:** 2015-09-01

**Authors:** Matteo Paci, Niccolò Landi, Gennaro Briganti, Bruna Lombardi

**Affiliations:** 1 Unit of Functional Rehabilitation, Department of Continuing Care, Azienda USL 4, Prato, Italy; 2 Private practice, Firenze, Italy; 3 IRCCS Fondazione Don Gnocchi, Firenze, Italy

**Keywords:** Randomised controlled trials, Quality ratings, Physiotherapy, Bibliometrics, Periodicals

## Abstract

**Background:**

Despite the use of citation rate as a measure of quality of research is strongly criticized and debated, it remain a widely used method to evaluate performances of researchers, articles and journals. The aim of this study was to test which factors are associated with citation rate of Randomised Controlled Trials (RCTs) published on the physiotherapy field.

**Methods:**

All RCTs abstracted in the Physiotherapy Evidence Database (PEDro), indexed in Scopus database and published in 2008 were included. PEDro score, language of publication, indexing in PubMed database, type of access to articles, subdiscipline, the number of authors, the country where the study was performed, the type of institution where the study was conducted and the number of centres involved in the study (multicentric vs single-centre). and the 2013 5-year impact factor of the publishing journals were considered as independent variables. Citation rate until December 2013 was extracted from Scopus database and used as dependent variable.

**Results:**

Six hundred and nineteen RCTs, published in 283 journals, were included and analysed. The 5-year impact factor was the strongest variable associated with the citation rate and explained approximately 50 % of the variance, and the number of authors explained an additional small part (about 1 %) of variability. The other variables were excluded from the model.

**Conclusions:**

The study highlights that 5-year Impact Factor, not accessibility (language of publication, indexing in PubMed database or the type of access to articles) or reported quality (PEDro score), is a strong predictor of the number of citations for RCTs in the physiotherapy field. Our findings support the increasingly widespread idea that citation analysis does not reflect the scientific merit of the cited work, at least in terms of reported quality.

The results of this study need to be confirmed with a publication window larger than one year.

## Background

The dissemination of research findings requires publication in peer-reviewed journals, and it is frequently measured through citation of the original work in subsequent publications. However, it is recognized that many factors outside the pure merit of the research or the authors influence such counts and many authors doubts that citation counts can really reflect the impact of scientific activity. The main reported sources of bias are self-citations, the increase in the total number of citations with time, or the correlation between the number of authors of an article and the number of citations it receives [1]. Moreover, citations are not necessarily representative of articles value, since papers can also be cited to be criticized.

A number of different indicators has been suggested to assess outcomes of biomedical research. In a recent review, Thonon et al. [2] found a total of 57 indicators aimed to assess research activity, collaboration, industrial production, dissemination and health service impact, in addition to scientific production and impact.

Anyway, citation analysis remain popular to evaluate performances of researchers, journals, universities, up to whole countries [3]. Citation count is also currently used as one of the criteria to select university professors in Italy [4] and for allocation of university research funds in many countries [5].

In addition, the number of citations received by an article is frequently viewed by researchers as the most useful measure of impact [6, 7]. It can be supposed that the number of citations received by an article should be related to its methodological quality and relevance of the issue. However, it also may be influenced by other factors, like accessibility to both database and full text, or language of publication.

A number of authors examined the citation rate of original researches in different fields of medicine [8–12]. However, articles with different study designs were included and, in most of the cases, the study design was found to be a strong predictor of citation rate [10, 11]. Randomised controlled trials (RCTs) are traditionally considered the gold standard for examining the efficacy of interventions. In the field of physiotherapy, RCTs represent a small part of the totality of research articles, but their rate is comparable to other disciplines [13–15]. The number of RCTs published in physiotherapy journals [14] and the quality of RCTs in physiotherapy field [16–18] show an increase over years. To our knowledge, no studies have been performed to specifically analyse the citation rate of this relevant type of articles.

The aim of this study was to test which factors are associated with citation rate of RCTs published within the physiotherapy field.

## Methods

All RCTs abstracted in the Physiotherapy Evidence Database (PEDro), indexed in Scopus database and published in 2008 were included. Article title, journal name, year of publication, ratings for each of the 11 items of the PEDro scale, and total PEDro score of all RCTs that had complete PEDro scale ratings were downloaded from PEDro database, a free database of RCTs, systematic reviews and clinical practice guidelines in physiotherapy. In addition to the PEDro score, the following variables were recorded for each article: (a) the language of publication (English vs other languages), (b) indexing in PubMed database, (c) the type of access to articles (open access, delayed open access or restricted access), (d) the subdiscipline; (e) the 2013 5-year Impact Factor of the journals where the articles were published; (f) the number of authors; (g) the country where the study was performed; (h) the type of institution where the study was conducted (academic vs non- academic) and (i) the number of centres involved in the study (multicentric vs single-centre).

Citation rate until December 2013 was extracted from Scopus database.

### Type of access

Access to the articles was defined as “open” when they were immediately available for free at the time of publication. Differently, they were defined as “delayed open” when they were available for free not immediately (e.g. after 6 months or 1 year). Finally, articles were considered as having “restricted access” when they were available only for subscribers.

### RCT Quality score (PEDro score)

The PEDro database provides the assessment of the reported methodological quality of each RCT using the PEDro scale [19, 20], which rates 11 aspects of methodological quality of RCTs as being either absent or present. The first item (eligibility criteria) is not scored because it does not relate to internal validity or statistical reporting, therefore the total score ranges from 10 (RCT that satisfies all criteria) to 0 (RCT that does not satisfy any of the criteria). The PEDro Scale total score has been shown to be reliable for use in systematic reviews of physical therapy RCTs [21]. There is evidence for its construct validity [22], convergent and construct validity [23], discriminative validity, face validity, and content validity [24].

### Subdisciplines

Subdiscipline classification was based on PEDro database classification. However, since a number of articles may be classified in different categories, clinical categories were preferred in classification process. For example, articles were included in Paediatrics or Gerontology categories only when the clinical problem addressed by the trials were not exactly recognizable.

### 5-year Impact factor

The 2013 5-year journal Impact Factor was extracted by Journal of Citation Report (JCR) (http://thomsonreuters.com/journal-citation-reports/). The 5-year Impact Factor is the average number of times articles from the journal published in the past five years have been cited in the JCR year. Therefore, 2013 5-year Impact Factor covers the period from 2008 to 2012.

### Country

When multicentric studies were performed in different countries, the country of the first author were recorded.

### Data analysis

Comparisons of proportions were analysed by the chi-square test, while the differences in the number of citations for each variable were compared with the one-way ANOVA, with Bonferroni post-hoc in case of variables with more than 2 categories.

Then, data were put in a stepwise multiple regression analysis with citation rate as dependent variable and language of publication, indexing in Pubmed database, type of access to articles, subdiscipline, PEDro score, number of authors, country, type of institution, number of centres and 2013 5-year Impact Factor of the publishing journals as independent variables.

In addition, the correlation between PEDro score and the 5-year Impact Factor was analysed with the Spearman’s correlation coefficient.

The level of statistical significance was set at 0.05. Data analyses were performed using the SPSS statistical package 12.0 for Windows.

## Results

A total of 674 articles were abstracted in PEDro database. Forty of them were not included in Scopus database and, within the remaining 634, other 15 were not scored in PEDro database. Then, 619 articles, published in 283 journals, were included and analysed. Trials were conducted in 37 countries with no difference in citation rate among them (*p* = .919). Articles were more frequently written in English language (93.7 %, *p* < .001), published with restricted access (58.5 %, *p* < .001), listed in PubMed (93.1 %, *p* < .001) and included in musculoskeletal subdiscipline (30.2 %, *p* < .001); 507 journals had 5-year Impact Factor and mean 5-year Impact Factor and PEDro score were 3.8 ± 5.5 and 6.1 ± 1.5, respectively.

The citation rate was significantly higher for articles written in English language, listed in PubMed, and published in journals with a 5-year Impact Factor (Table 1). Post-hoc analyses showed that the citation rate was significantly higher for articles published with delayed open access (vs open access: *p* < .001; vs restricted access: *p* = .042) while no significant differences were found between subdisciplines.

**Table 1:** Comparisons of citation rate among articles’ characteristics (*n* = 619)

Variable		frequency	citations	*p* value
Language				< .001
	English	580 (93.7 %)	28 ± 41	
	Others	39 (6.3 %)	3 ± 4	
Subdiscipline				.023
	Musculoskeletal	187 (30.2 %)	22 ± 21	
	Neurology	91 (14.7 %)	26 ± 28	
	Cardiothoracics	89 (14.4 %)	36 ± 69	
	Pediatrics	18 (2.9 %)	19 ± 11	
	Gerontology	52 (8.4 %)	31 ± 56	
	Continence/womens’ health	25 (4.0 %)	16 ± 14	
	Oncology	29 (4.7 %)	28 ± 20	
	Sport	11 (1.8 %)	28 ± 24	
	Occupational health	8 (1.3 %)	16 ± 9	
	Endocrinology	68 (11.0 %)	38 ± 57	
	Others	41 (6.6 %)	14 ± 12	
Pubmed				< .001
	yes	576 (93.1 %)	28 ± 41	
	no	43 (6.9 %)	5 ± 5	
Countries				.919
	Australia	50 (8.1 %)	34 ± 58	
	Canada	26 (4.2 %)	44 ± 84	
	United Kingdom	57 (9.2 %)	24 ± 35	
	USA	137 (22.1 %)	32 ± 34	
	Sweden	26 (4.2 %)	19 ± 14	
	Brazil	29 (4.7 %)	16 ± 14	
	Germany	25 (4.0 %)	19 ± 19	
	Turkey	26 (4.2 %)	23 ± 23	
	The Netherlands	35 (5.7 %)	33 ± 37	
	China	41 (6.6 %)	19 ± 58	
	Others	167 (27.0 %)		
Type of access				< .001
	Open access	79 (12.6 %)	34 ± 56	
	Delayed open access	175 (28.9 %)	37 ± 59	
	Restricted access	365 (58.5 %)	20 ± 18	
Number of centres				< .001
	multicentric	532 (85.9 %)	26 ± 1	
	single-centre	87 (14.1 %)	83 ± 9	
5-year Impact Factor				< .001
	Yes	507 (81.9 %)	29 ± 43	
	No	112 (18.1 %)	12 ± 16	
Academic setting				.965
	Yes	455 (73,5 %)	26 ± 36	
	No	164 (26.5 %)	26 ± 49	

The multiple regression analysis showed that the 5-year Impact Factor was the strongest independent variable associated with the citation rate (β = 4.828; 95 % confidence interval = 4.382; 5.276), explained approximately 50 % of the variance (adjusted R^2^ = .503). The number of authors also was independently associated with citation rate (β = 1.700; 95 % confidence interval = .894; 2.506), and explained approximately further 1 % of variability (cumulative adjusted R^2^ = .516). The other variables were excluded from the model (Table 2).

**Table 2:** Regression analysis (dependent variable: citation rate)

	Beta	*t*-test	*P* value
Model:			
Constant		-.679	<.001
5 year Impact Factor	.658	21.276	<.001
Number of authors	.128	4.144	<.001
Excluded variables:			
PEDro score	.030	1.034	.302
Country	.011	.390	.697
Academic	-.005	-.162	.871
Multicentric	.041	1.321	.187
Language	.029	.999	.318
Subdiscipline	-.017	-.608	.544
Pubmed	.034	1.191	.234
Type of access	-.027	-.907	.365

A significant correlation between PEDro score and 5-year Impact Factor of the publishing journal was found (Spearman’s correlation coefficient = .197; *p* < .001).

## Discussion

This study was aimed to test what factors are associated with citation rate of RCTs published on the physiotherapy field.

Evaluating scientific quality of research groups and scientific journals is a notoriously difficult problem that has no accepted standard solution. The use of citation frequency as a measure of quality of research is strongly criticized and debated [25, 26]. Anyway, the growing competition for research funding and academic positions, combined with an increasing use of bibliometric parameters to evaluate careers (e.g. number of publications and the impact factor of the journals they appeared in), pressures scientists into continuously producing publishable and citable results [27]. Also Journals and Editors are interested in bibliometric parameters because they may influence subscriptions and where authors submit papers [28, 29].

In this work, we considered as independent variables many of the most relevant factors that may reduce the value of citation count as indicator of scientific impact [1].

We found that the 5-year Impact Factor of the original publishing journal was the strongest predictor of citations. This result was not fully expected, since 5-year Impact Factor is a bibliometric indicator for journals, while the citation rate is for individual papers. In fact, Shadgan et al. [30] found that the most frequently cited articles published in the rehabilitation field were not always in the journals with the highest Impact Factor. Also Lozano et al. [31] found that, since 1990, the proportion of highly cited papers coming from highly cited journals has been decreasing, and accordingly, the proportion of highly cited papers not coming from highly cited journals has also been increasing. In addition, the 5-year Impact Factor was extracted from JCR, while the number of citations per article was extracted from Scopus and different citation databases may produce different citation count [32]. Then, these two variables cannot be judged co-dependent, since they are placed on different levels and based on two different databases: an article may be published in a journal with high bibliometric indicators and obtain few citations and, at the same time, a paper published in a journal not indexed in JCR may receive high number of citations within Scopus database. Since PEDro has been shown to be the largest database on physiotherapy field [33, 34], we chose to use Scopus database for the citation count because it includes a larger number of journals (and articles) in comparison to JCR [35], the two most widely used tools for generating citation count. A different option could have been Google Scholar, but its use is largely criticized because it covers and uses for citation count, in addition to print and electronic journals, conference proceedings, books, theses, dissertations and abstracts (i.e. not peer-reviewed literature) [32, 35]. Furthermore, when searching for documents citing a specific paper in Google Scholar, a few duplicates can be found because the same article can be extracted from different archives [36].

Callaham et al. [8] found similar results in a study on articles published in the field of Emergency Medicine and indexed in PubMed. In a cohort of 204 published articles, the strongest predictor of citations per year was the Impact Factor of the original publishing journal. Our results suggest that citation may be more strongly influenced by the reputation of the publishing journal than by the quality of research or its accessibility (i.e. electronic searching, language of publication, open access system). This is a well known phenomenon, similar to what Cozzens [37] calls “success-breeds-success” or also known as “Matthew Effect” [38], which is possible not only for highly-cited scientists, but also for highly-cited publications [39].

A different explanation might be that journals with high 5-year Impact Factor publishes high quality studies in any case. This seems to be questionable, since we found a weak correlation between quality (total PEDro score) of RCTs and 5-year Impact Factor of the publishing journal. Costa et al. [40] found no correlation between RCTs quality on physiotherapy interventions, assessed by the PEDro score, and the 2-year Impact Factor of journals where the studies were published. On the basis of this investigation, Authors concluded that the journals 2-year Impact Factor is not a valid measure of RCT quality. Thus, the journal where a paper is published seems to give it more chances to be cited over and above their intrinsic quality or relevance [39].

In previous studies the citation count was performed after 3 [10], 3,5 [8] or 4 years [41] from publication. A period of 5 years may be better suited, especially for clinical journals, for which there is usually a relatively high citation rate up to at least 6 years after publication or even longer [42]. For this reason, the 2013 5-year Impact Factor was chosen as bibliometric indicator. We have to take into account that journals included in JCR can change over time: for example, within the category “Rehabilitation”, 28 journals were included in the year 2008 (year of publication of RCTs) and 62 in the year 2013 (year of final count of citations).

More surprisingly, methodological quality of RCTs, assessed by PEDro score, was not a predictor of citation rate. Items of PEDro scale assess the internal validity and statistical reporting of articles, while factors related to external validity (e.g. study participant recruitment and selection procedures, choice of outcomes measures) [43] are not included in the scale. The choice of using this type of scale might have played down the weight of the quality of RCTs as predictor in the model. Quality is a complex concept not easy to measure. For example, it has been shown that the citation counts of papers were highly related to clinical utility [44, 45].

In our sample, open access system was not a predictor of citation rate. This result is not really surprising, since the real role of the free access to journal articles on their citation rate is still debated. For example, in most studies, it has been suggested that free access articles are cited significantly more times than articles that are not open access [46, 47]. On the other hand, others [48, 49] showed that open access articles received significantly more downloads and audience, but they were not cited more frequently than non-open access articles. In the same way, PubMed indexing, subdisciplines, English language were not predictors of citation rate and this is explainable by the high number of articles having these characteristics (about 95 %). PubMed database was chosen as independent variable because it is free and provide open access to the records to all interested clinicians, researchers, and trainees and also to the public in general. The association of PEDro score with language of publication and subdisciplines were recently investigated. Despite language of publication (English versus other languages) has been found to be associated with the methodological quality of reports of physiotherapy trials, the magnitude of this influence is small [50]. It has also recently found that methodological quality of trial reports varies according to the subdiscipline of physiotherapy, classified according to the PEDro database classification [17]. The country and the type of institution where the study was performed and the number of centres involved in the study also were not associated to citation rate. Otherwise, Pasterkamp et al. [51] observed that citation frequency may be significantly augmented by nation oriented citation bias. Many of the these variables (open access system, language of publication, subdisciplines, country) are commonly considered sources of bias in using citation rates as a measure of scientific impact [1] and their exclusion from the regression model suggests that their influence is low in our sample.

As found by Beaver [52], the number of authors was associated to citation frequency and it is also considered a sources of bias. However, in our analysis it was a very poor predictor of citation rate.

The study had some limitations, that should be taken into account in the interpretation of results. About 8 % (*n* = 55) of articles were excluded. The use of Scopus may have introduced bias. However, in our sample, Scopus failed to list less than 6 % of trials found in PEDro, and such coverage can be considered acceptable, since PEDro have virtually complete coverage of RCTs in physiotherapy field [33, 34]. In addition, Fell et al. [53] reported that Scopus provide a strong coverage of physical therapy literature. Fifteen articles (about 2 %) were not scored in PEDro database and excluded from the study because the full text are written in languages that we were not able to translate. Articles published in just one year were included. We know that IF can vary from year to year depending on the published material in a particular year; in fact, a large random component in IF changes has been reported [54].

The inclusion of self-citations is a potential source of bias. However, the exclusion of self-citations is a controversial issue. In fact, one should discriminate between different kinds of author self-citations, from those that are informative to those that are self-enhancing [55].

Finally, a number of variables, such as clinical relevance or newsworthiness, as well as generalisability of results, were not included in the model because they are hard to measure.

## Conclusions

In conclusion, the study highlights that neither accessibility (language of publication, indexing in PubMed database or the type of access to articles) nor reported quality (PEDro score), but 5-year Impact Factor, is a strong predictor of the number of citations for RCTs in the physiotherapy field. Our findings support the increasingly widespread idea that citation analysis does not reflect the scientific merit of the cited work, at least in terms of reported quality. In our sample, the main threat is the so called “Matthew Effect”.

The results of this study need to be confirmed by studies on a publication time longer than one year.

## Abbreviations

RCT: Randomised controlled trial
JCR: Journal citation report
PEDro: Physiotherapy evidence database
